# Mento’s change model in teaching competency-based medical education

**DOI:** 10.1186/s12909-019-1896-0

**Published:** 2019-12-27

**Authors:** Yajnavalka Banerjee, Christopher Tuffnell, Rania Alkhadragy

**Affiliations:** 1 College of Medicine, Mohammed Bin Rashid University of Medicine and Health Sciences, Dubai Health Care City, Dubai, United Arab Emirates; 2 Centre for Outcomes and Research in Education, Mohammed Bin Rashid University of Medicine and Health Sciences, Dubai, United Arab Emirates; 3 Centre for Medical Education, University of Dundee, Dundee, UK; 4 Department of Medical Education, Faculty of Medicine, Suez Canal University, Ismailia, Egypt

**Keywords:** Change-management, Kotter’s model of change, Mento’s model of change, Flipped-teaching, Active learning, Competency based medical education, 6D-approach, Leadership theory

## Abstract

**Background:**

Resistance to change is customary and is expected in any organization. However, most of the downsides of change can be avoided if the organization/individual prepares for the change by acknowledging guided strategies. In healthcare, change is the state of nature, which has also translated to medical education (ME). ME in the current era has undergone a shift from a traditional content-based curriculum to a competency-based curriculum. Recently, however, the broader social-accountability movement has accelerated this rate of transformation. One of the key challenges to educators harbingering this transformation to competency-based medical education (CBME) is to redesign the processes of teaching.

**Aim:**

Here we define a framework designed using Mento’s model of change that will totally agree with introducing positive change in teaching in an institution undergoing transformation from a traditional content-based curriculum to a competency-based curriculum.

**Methodology:**

Using Schein’s “unfreezing” as a guide term we critically reflected on the popular change-management models, to home in on Kotter’s model of change to transform organizations. However, Kotter’s change-model draws from Situational and Contingency Leadership Theories, which may not agree with academic organizations involved in ME. As such organizations adhere to Transactional and Transformational Leadership archetypes, where Leadership is constructively executed by “The Leader Team”, we decided to adopt Mento’s change-model for our study. Mento’s model not only draws from the precepts of Kotter’s model, but also incorporates axioms of Jick’s and GE’s change-models.

**Results:**

Using Mento’s model a framework was blueprinted to implement active learning (AL) strategies in CBME. Here we have elaborated on the framework using the exemplar of flipped teaching. The development of this framework required the design and execution of a faculty development program, and a step by step guidance plan to chaperon, instruct and implement change in teaching to harbinger CBME. Further, we have also reflected on the change process using Gravin’s framework.

**Conclusion:**

To our knowledge this is the first report of the use of Mento’s model of change in medical education. Also, the blueprinted framework is supported by acknowledged leadership theories and can be translated to implement any curricular change in CBME.

## Background

## Introduction

Implementing change in any organization is a challenging feat. In the words of management thought leader and business entrepreneur John Kotter “Why change is so hard? Because in order to make any transformation successful, you must change more than just the structure and operations of an organization – you need to change people’s behaviour. And that is never easy” [1]. Resistance to change is customary and is expected in any organization. However, most of the downsides of change can be avoided if the organization/individual prepares for the change by acknowledging guided strategies.

In health care, change is the state of nature, and this has also translated to medical education [2]. Medical education (ME) in the current era has undergone Flexnerian revolution i.e. a shift from a traditional content-based curriculum to a competency-based curriculum [3]. The latter is defined “as a form of education that derives a curriculum from an analysis of prospective or actual role in modern society and attempts to certify student progress on the bases of demonstrated performance in some or all of that aspects of the role” [4–6]. Within ME, the so-called transformation from traditional content-based curriculum to a competency-based curriculum has been underway for the last five decades [7]. Recently, however, the broader social-accountability movement has accelerated this rate of transformation. As best described by Sullivan [8], accreditation bodies now envisage health-professionals to demonstrate that they are truly achieving what they set out to do. However, this sudden fast-tracked transformation has presented ***three KEY challenges*** to educators harbingering competency-based medical education (CBME) [9]:

I. Systematising the structural changes that will be necessary to deliver new curricula and methods of assessment.
II. Redesigning and amending the processes of teaching and evaluation.
III. Facilitating to change the ethos of education, so that CBME paradigm gains acceptance.

*This paper focuses on defining a framework that will totally agree with introducing positive change in teaching in an institution undergoing transformation from a traditional content-based curriculum to a competency-based curriculum.*

### Teaching needs in CBME

In CBME the learner doesn’t just acquire knowledge to be ready at the time of final examination, instead knowledge is acquired and assessed throughout a continuum of learning. In other words, CBME is a learner-centred, active, and lifelong learning experience. In this regard, reform efforts supporting the shift to CBME in medical education have emphasized and advocated the importance of active learning (AL) to advance student engagement and critical thinking skills [10, 11].

AL can be defined as an all-encompassing expression that comprises of a range of teaching and learning techniques [12]. AL embodies a change from the traditional “sage on the stage” method of teaching that has a predisposition to render learners bored or passive [12]. In AL, the learners take responsibility for their learning by participating and engaging in diverse in-class exercises. This teaching strategy therefore accentuates higher-order thinking [13, 14] and problem-solving skills [15, 16] in the learner. In fact, AL strategies in medical education have been found to augment learning [17, 18], engagement [19–21], peer collaboration [22, 23] and evidence-based medicine [24]. However, despite the obvious rewards of AL there is a severe dearth in its adaptation/implementation in medical education. In fact, a growing body of literature still bemoans the lack of AL strategies in medical education [25–28]. At issue is the absence of a framework to implement a “pedagogical change” that will encourage, implement and nurture AL.

*In summary, this paper aims to define and elaborate on a framework, designed using Mento’s 12-step change-management model, to initiate change in pedagogy to implement AL strategies in a competency-based medical curriculum. In designing the framework, the exemplar of flipped-teaching model has been used. However, the designed framework can be adapted to implement and sustain any AL strategy or a specific change in any competency-based medical curriculum.* Undeniably, this is a baby step in the development/improvement of AL paradigms. Nevertheless, the framework provides the primer to initiate a pedagogical transformation in CBME, which will facilitate the founding of a guidance plan towards an effective pedagogical change.

### Choosing the change-management model

As best stated by Senge in The Fifth Discipline [29] “*We both fear and seek change. Or as one seasoned organizational change consultant once put it, People don’t resist change. They resist being changed.*” Therefore, the key challenge we faced was to decide on a model/framework for blue-printing the change-strategy. Added to this challenge was to apply the framework to address the so-called “Universal Challenges” one encounters in any learning organization. Senge discusses these challenges extensively in his book *The Dance of Change: The Challenges of Sustaining Momentum in Learning Organizations* [30]. Briefly, these challenges can be classified into three categories: I. challenges of initiating change; II. challenges of sustaining momentum; and III challenges of system wide redesign and rethinking (Refer to Table 1 for details).

**Table 1:** Senge’s classification of challenges in organizational change

**The Challenges of Initiating:** ➢ Not Enough Time – “We don’t have time for this stuff!” ➢ No Help (Coaching and Support) – “We have no help!” “We don’t know what we’re doing!” ➢ Not Relevant – “This stuff isn’t relevant!” ➢ Walking the Talk – “They’re not walking the talk!”
**The Challenges of Sustaining** ➢ Fear and Anxiety – “This stuff is _________.” (Am I safe? Am I adequate? Can I trust others? Can I trust myself?) ➢ Assessment and Measurement – “This stuff isn’t working!” ➢ True Believers – “They don’t understand us!” / “We have the right way!” **AND** ➢ Non-Believers – “I have no idea what these people are doing!”/ “They are acting like a cult!”
**The Challenges of Redesigning and Rethinking** ➢ Governance – “They won’t give up the power!”/ “Who’s in charge of this stuff!” ➢ Diffusion – “We keep reinventing the wheel!” ➢ Strategy and Purpose – “Where are we going? What are we here for?”

In line, when deciding on a change-management model for blue-printing the change-strategy, we focused on FOUR well endorsed models of change, which addressed most of the challenges in Table 1. These FOUR models are (1) Schein’s Steps of Change [31]; (2) Kotter’s 8-steps to transform organization [1, 32]; (3) Senge’s – Challenges of Change [29]; and (4) Fullan and Miles’ propositions for success [33]. The key aspects of these change-management models are indicated in, Table 2.

**Table 2:**
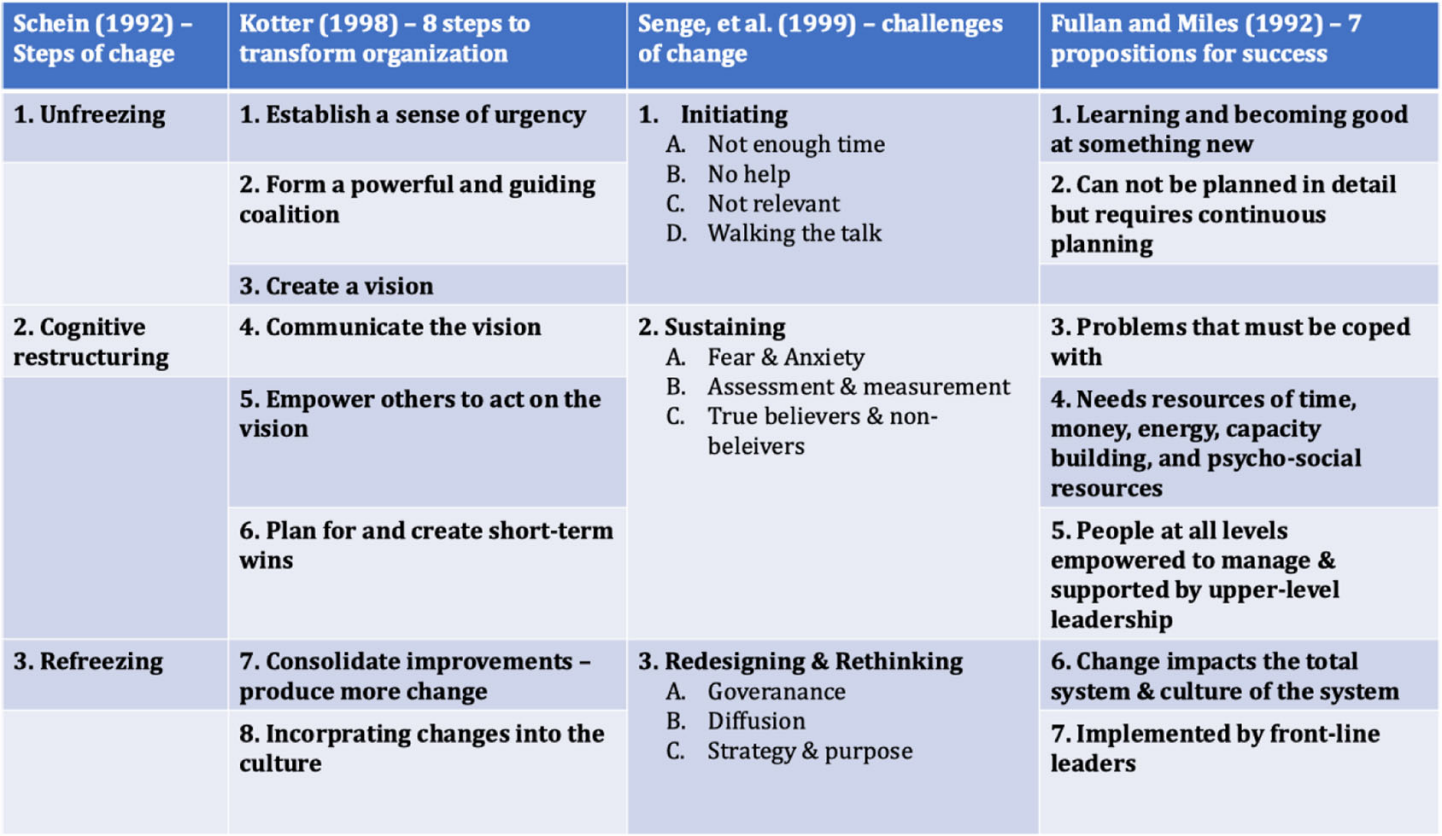
The different change-management models considered while pursuing the current study

Next, employing Schein’s “unfreezing” as a guide term (Table 2), we critically reflected on the other change-management models (Table 2), where we found that the 8 Steps to Transform Organizations of Kotter would cause change of thought or “unfreezing”; institute a wisdom of urgency; form a commanding and guiding coalition; and create a vision.

In line, based on the above cogitation, our initial urge was to employ Kotter’s model. But, when we compared architectural framework of Kotter’s model in light of the different leadership theories, it flaunted one flaw. The leadership style requiring the execution of Kotter’s model draws from Situational and Contingency Leadership Theories [34], whereas an academic institution functions best through Transactional and Transformational Leadership archetypes [35, 36], where Leadership is constructively executed through a so-called “informal kibitzing” from a team of experts “The Leader Team”.

**Fig. 1:**
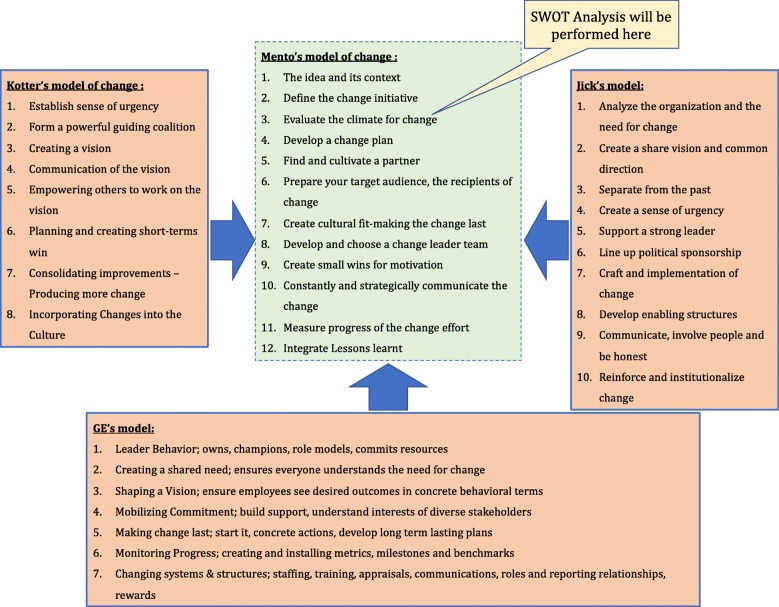
Change management plan of Mento, which was used in this project. The plan is a blend of three popular models (*Refer to text for references and details*). The step of the plan where the Leader Team pursued a SWOT (*strengths, weaknesses, opportunities, and threats*) analysis is indicated using a callbox. (*Note: The rationale for adapting Mento’s model in this project is discussed in text*)

Using “The Leader Team” as a search term, we were able to identify Mento’s model of change [37], which draws from the precepts of Kotter’s model, but also incorporates axioms from two other model of change: Jick’s model [38] and GE’s model [39] (Fig. 1). Although not a very well-known change model, especially in the domain of medical education and healthcare, after carefully comparing the overall designs of Kotter’s and Mento’s models of change, we decided to adapt and implement Mento’s model for our study. In fact, this change model was developed and successfully implemented in a fortune 500 defence industry firm, with positive outcomes [37].

### Why MENTO’S and not KOTTER’S

Initially, when deciding on the model of change we conducted a cursory search with the search-term “Kotter’s” in PubMed, which retrieved 10 articles [32, 40–48]. When we conducted a similar search with the search term “Mento’s” it did not retrieve a single article in the domain of healthcare change-management. In fact, Kotter’s model of change is by far the most popular change-model in the healthcare milieu. However, while considering a change-model for this project, we chose Mento’s model, over Kotter’s, as there are certain drawbacks with Kotter’s change-model, which have been befittingly addressed in Mento’s model, making it more pertinent and capable for academia. *Also, to our knowledge this is the first report of the use of Mento’s model of change in medical education*.

**Fig. 2:**
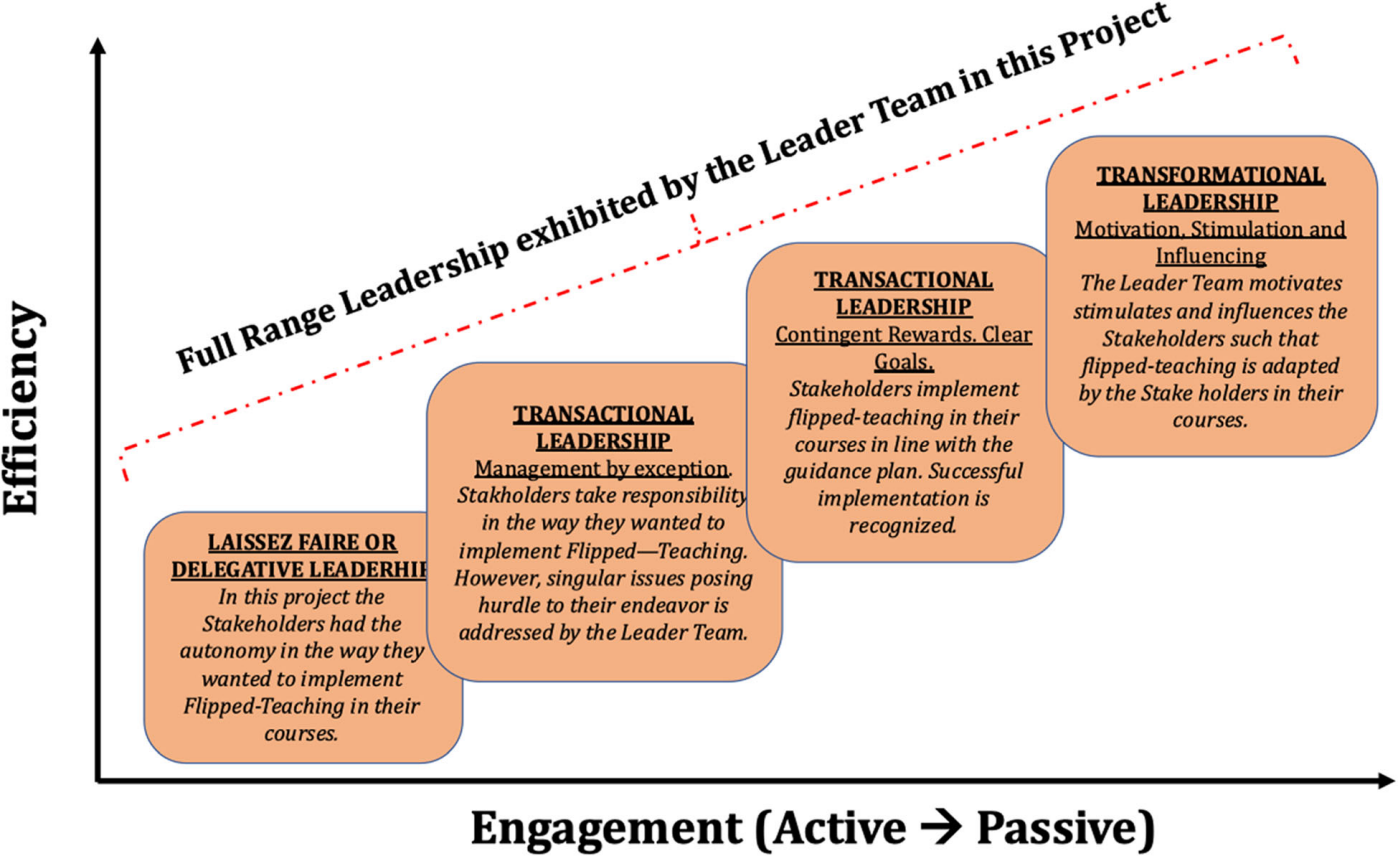
Full Range Leadership Model elaborating the Leader Team’s role in implementing change using Mento’s change-model. (Note: *The Leader Team exhibited both Transactional and Transformational Leadership, as well as allowed the stakeholders to express their independent thoughts and concepts (*Laissez-Faire *Leadership))*

A. The eight-step change model of Kotter’s, doesn’t delegate a step towards preparing the recipients of change i.e. the stakeholders for the change process. However, *Step 6 of Mento’s model takes care of this aspect* (Fig. 1).
B. Leadership aspect of Kotter’s model does not include the informal organisation, thus perpetuates the top-down style of consolidated command and control leadership. Mento’s model on other-hand proposes to organize a “Leader Team” which can provide better guidance than an individual leader, as the “Leader Team” can be carefully assembled to maximize the appropriate skill sets (Fig. 2).
C. One of the key aspects missing in Kotter’s model is the measurement of the progress of change effort, *dealt in Step 11 of Mento’s model* (Fig. 1). This step allows the Leader Team to track the progress of the change effort, and avail suitable measures to tackle encountered hurdles.
D. Any change effort is often met with resistance from people in the organization [37, 49, 50], an aspect which Kotter’s model fails to take into consideration. Mento’s suitably addresses this aspect at two levels: *Steps 6 and 10* (Fig. 1) [37].

## Main text

### Employing MENTO’S model to implement flipped teaching

#### Prelude to change

The flipped-model of teaching is beneficial for knowledge gain in undergraduate medical education (UME) [51–54]. In this teaching-model, corpus of didactic material is consumed by the learner at home at their own time and pace. In-class time is focused on application, simulation, case-based discussion, or problem solving [55–57]. Because such a pedagogical method facilitates active-learning and is grounded in social constructivism, medical education experts advocate this teaching-model, leading to health professions schools to adopt this approach in pre-clinical, clinical, and graduate medical education [58].

**Fig. 3:**
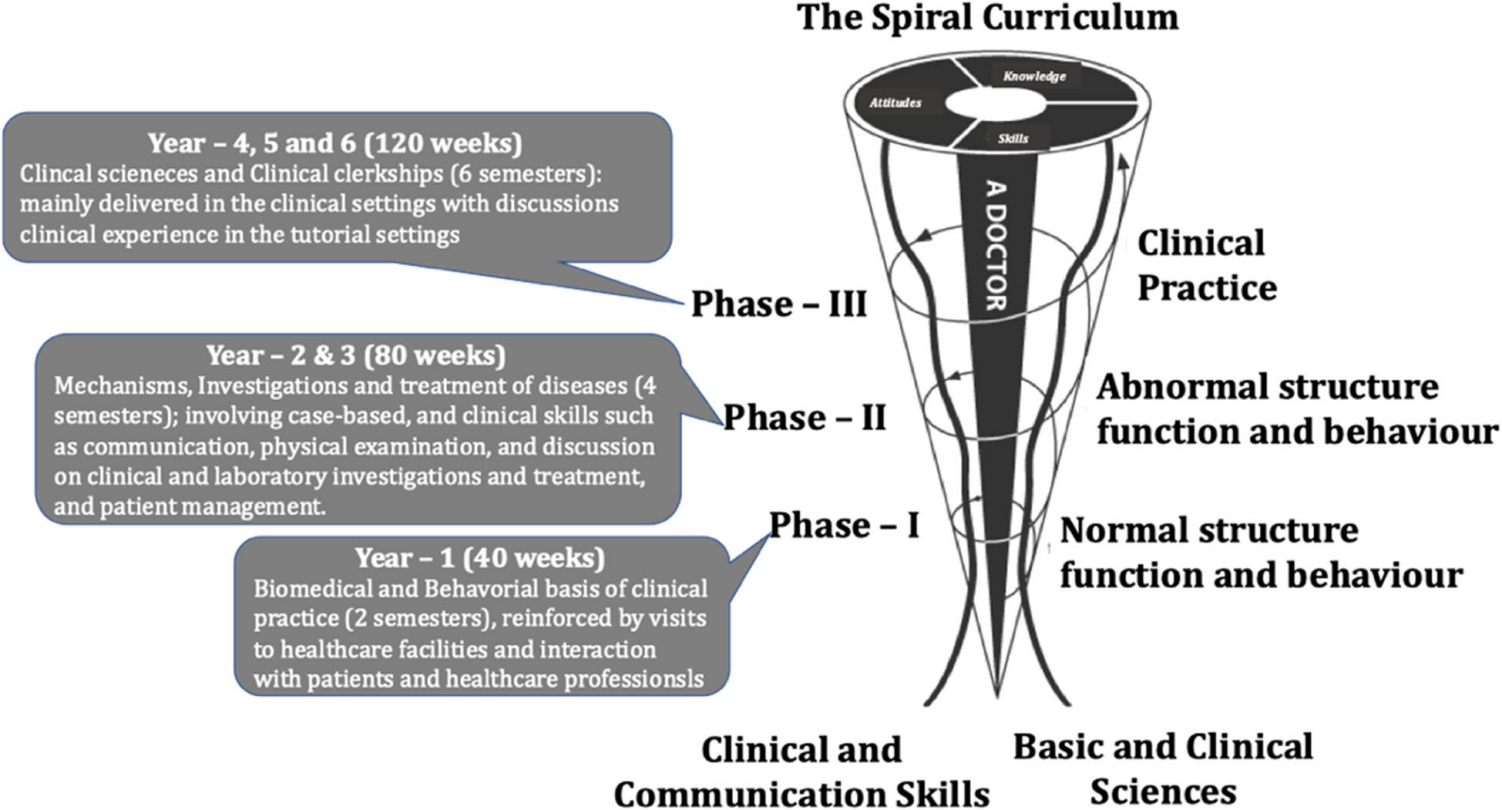
The undergraduate medical curriculum at Mohammed Bin Rashid University of Medicine and Health Sciences. The curriculum is divided into three phases and spans over 6 years. Each phase of the undergraduate medical curriculum includes integrated courses and builds on the preceding one, such that the curriculum is a “spiral,” and the students repeat the study of a subject, each time at a higher level of difficulty and in greater depth

MBRU is a new medical school located in Dubai Health Care City (DHCC), the health care hub of UAE, with an undergraduate entry medical program, where the curriculum is founded on a competency-based educational model, and spans over six years. The MBRU curriculum is divided into 3 phases (Fig. 3) [59]. Each phase of the MBBS curriculum includes integrated courses and builds on the preceding one, such that the curriculum is “spiral”, and the students repeat concepts pertaining to a subject, where with each successive encounter, concepts build on the previous one (Fig. 3). The school has a diverse student population, drawing students from more than 19 countries across the globe. Approximately, 75% of the students are females [59].

**Fig. 4:**
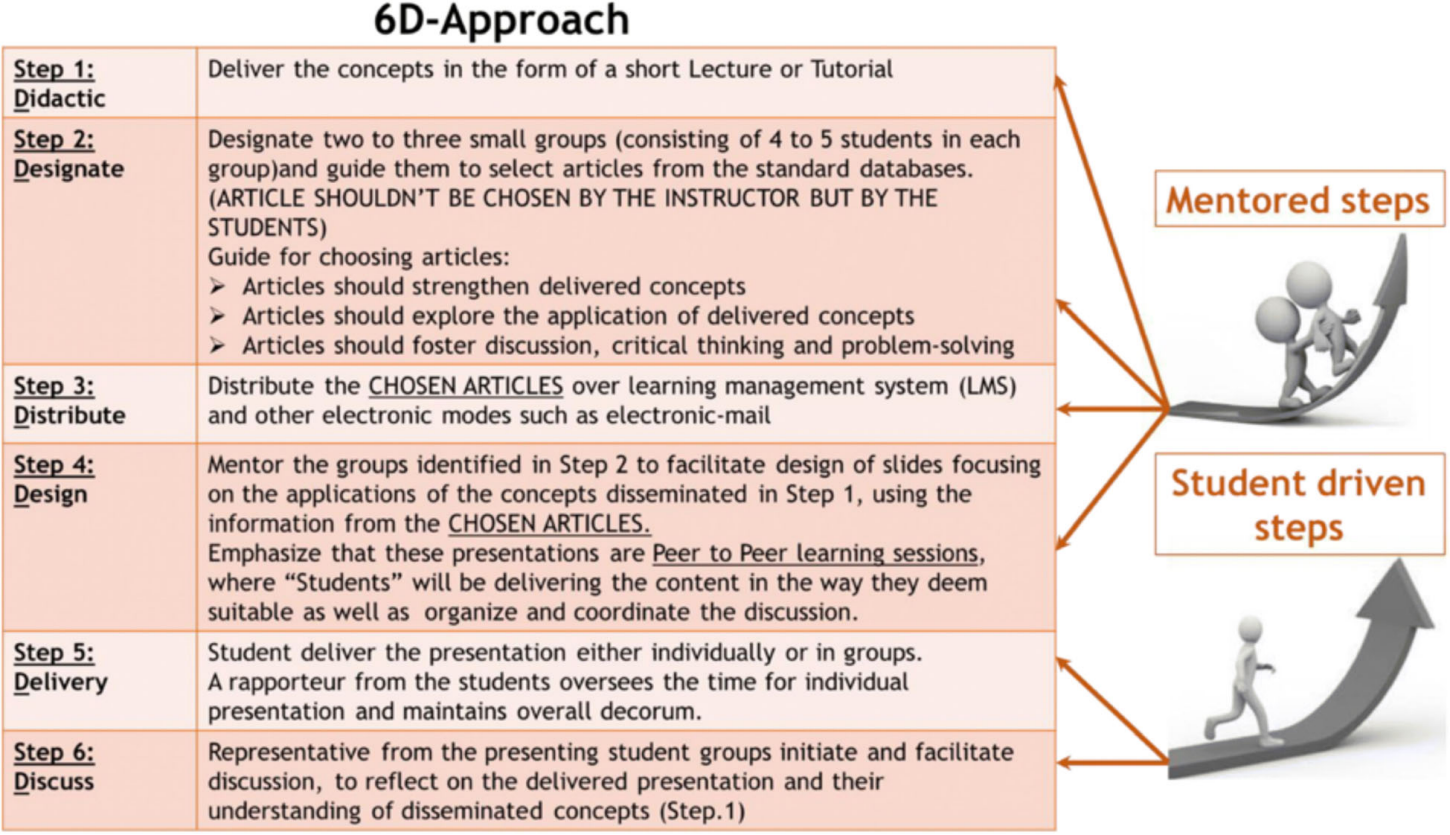
The different steps of the 6D-Approach. (*The initial steps are mentor dependent, whereas the concluding steps are student driven)*

The flipped-model of teaching was implemented in delivering the sessions of the three-credit Molecular Biology and Principles of Genetics module in phase-1, semester-1 of the MBBS curriculum at MBRU [60], for the student cohort 18–19. Briefly, we availed a novel 6D-Approach where mentored journal clubs were employed for the dissemination of complex concepts in molecular biology as shown in Fig. 4, [60]. The 6D-approach was positively received by the students and the formal feedback for the course: Molecular Biology and Principles of Genetics, where the approach was repeatedly employed, indicated that students expressed satisfaction with the teaching strategies employed in the course, with ~ 89% of the students in the cohort strongly agreeing with the highest grading score “extremely satisfied”. Further, the flipped-approach through the use of mentored journal clubs encouraged retention of knowledge, critical thinking, metacognition, collaboration and leadership skills in addition to self-evaluation and peer feedback.

Student cohort 18–19 also suggested that the flipped-teaching model should be implemented in other Phase-1 courses especially the structure-function courses, where the bulk of anatomy and physiology is delivered. These courses provide the scientific basis of medical practice, where students gain knowledge about each body system, focusing on the mechanisms of cellular structure and function.

In line, the current project aimed at introducing a change in pedagogical methodology through the implementation of flipped teaching, using Mento’s model of change framework, in structure-function courses (Table 3), of the MBBS curriculum. The different steps of the change plan are shown in Table 4.

**Table 3:**
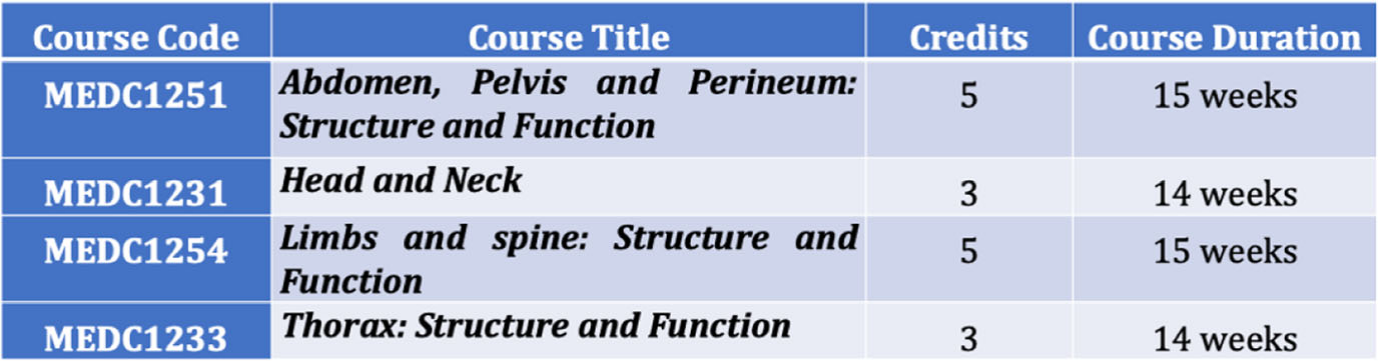
The Phase − 1 structure-function courses where flipped- teaching is to be implemented

**Table 4:** Guidance plan showing the activities and timeline corresponding to each step of Mento’s Change Model

Step No.	Steps of Mento’s Model of Change	Activity to facilitate/implement the change	Timeline
1	**The idea and its context**	Preliminary results from the phase-1 semester-1 course of MBPG showed that flipped-model of pedagogy facilitates better learning in UME. *The idea is to integrate flipped-teaching in the phase-1 semester-2 structure-function courses (table-2)*.	N/A
2	**Define the change initiative**	Present to the concerned stakeholders: ⇒ What is flipped-teaching? ⇒ Benefits of flipped-teaching. ⇒ Successful case-studies of flipped teaching (literature review).	FOUR-weeks prior to course initiation
3	**Evaluate the climate for change**	Appraise the necessary resources, prior knowledge of stakeholders and technological know-how required to successfully implement flipped-teaching in the structure-function courses, *through SWOT analysis*.	FOUR-weeks prior to course initiation
4	**Develop a change plan**	Work with technology-enhanced learning (TEL) team at MBRU to develop a faculty development plan to train the stakeholders regarding strategies to implement flipped-teaching in structure-function courses.	THREE-weeks prior to course initiation
5	**Find and cultivate a sponsor**	Schedule meetings with MBRU academic leadership (**Dean/Associate Deans/ Departmental Chairs**) to inform them about the benefits of flipped-teaching and the resources required.	THREE-weeks prior to course initiation
6	**Prepare your target audience**	⇒ Organize faculty development workshops in collaboration with the TEL team to inform stakeholders about “how” to implement flipped-teaching structure-function courses. ⇒ Circulate nano-lectures on of flipped-teaching to stakeholders over WhatsApp.	TWO-weeks prior to course initiation
7	**Create the cultural fit**	Create linkage between students’ learning approaches and flipped-teaching to elaborate to the concerned stakeholders “why” there is a necessity to create a culture of innovative pedagogy in UME.	TWO-weeks prior to course initiation
8	**Develop and choose a leader team**	Create an informal “Leader Team” consisting of course-director and instructors from the MBPG course, such that they can guide and encourage the stakeholders to implement flipped-teaching in their courses. (*at least NINE flipped-teaching sessions over FIVE weeks*)	ONE–FIVE weeks into the course
9	**Create small wins for motivation**	Identify the stakeholders who successfully integrated flipped teaching in their courses and request them to present their experiences in this effort to the MBRU academic leadership and other concerned stakeholders.	FOUR-FIVE weeks into the course
10	**Constantly and strategically communicate the change**	During the whole transformation process: ⇒ Create a “Learning community” such that stakeholders can learn from each other about strategies to successfully implement flipped-teaching in pedagogy. ⇒ Try to address hurdles that are faced by stakeholders in their endeavor, by communicating the change process to Sponsors	ONE–FIVE weeks into the course
11	**Measure progress of the change effort**	⇒ Refer to the updated pedagogical techniques of concerned courses to appraise the number to teaching sessions where flipped-teaching was implemented. ⇒ Evaluate the attitude of stakeholders towards flipped-teaching following the transformation initiative using ADKAR framework. ⇒ Assess the performance of the students in these courses to identify if flipped-teaching was beneficial over traditional method. ⇒ Conduct student feedback to assess the perception of students towards flipped teaching.	SIXTH-week into the course following the Mid-term assessments
12	**Integrate lessons learned**	Using a reflective-framework conduct an After Action Review to: ⇒ Map the transformation process ⇒ Identify hurdles that further required to be tackled such that flipped-teaching can be successfully integrated in other courses.	SIXTH-week into the course following the Mid-term assessments
	PREPARATORY TIME FOR IMPLEMENTING THE TRANSFORMATION		FOUR-WEEKS
	TIME REQUIRED FOR IMPLEMENTING/ASSESSING THE TRANSFORMATION		FIVE-WEEKS
	TOTAL STUDY DURATION (PREPARATION + IMPLEMENTATION + ASSESSMENT)		NINE-WEEKS

#### Mento’s step 1: the idea and its context

In order to initiate a change, it is pivotal to define the idea which needs to be changed and ascertain the change vision. In this project in order to define the change Senge’s conceptual framework of *creative tension* was employed [29]. Creative tension evolves from clearly recognizing where we want to be, the vision, concurrently acknowledging our current status i.e. where we are now, the so-called current reality. Implementation of creative tension facilitated us to recognize:

A. ***The Current Reality***, where it was observed that in all Phase-I courses of the MBBS curriculum at MBRU (except Molecular Biology and Principles of Genetics (MBPG)) the instructors employed traditional “Sage on the stage” technique to disseminate the session/course learning objectives.
B. ***The Vision***, which was to successfully integrate flipped-teaching in the phase-1 structure-function courses (Table 3). Therefore, this project can be categorized as a proof-of-concept study, where the key purpose is delineating a framework, which later can be applied to other courses in Phase-I as well as other Phases in the MBBS curriculum at MBRU.

#### Mento’s step 2: define the change initiative

This step tracks closely with step-1 of Jick’s change model [38] (Fig. 1) and purposes to define the protagonists of the key players in all change efforts: *Strategists, Implementers and Recipients or Stakeholders*.

A. ***Strategists*** are responsible for the initial work, which encompasses identifying the need for change, creating a vision of the desired outcome, deciding what change is feasible, and deciding who should sponsor and defend it. The strategist-group consisted of the course director and the lead instructor of MBPG.
B. ***Implementers*** shape, empower, orchestrate and facilitate successful progress in the change process. The implementer group included course director and the lead instructor of MBPG, as well as a digital education advisor from Technology Enhanced Learning (TEL) department of MBRU.
C. ***Recipients or stakeholders*** represent the largest group of individuals that must adapt/acclimatize to the change. The course director and lead instructors from each of the structure-function courses offered in Phase-1 (Table 3) in the MBBS curriculum at MBRU, consisted of the recipient group for this study.

#### Mento’s step 3: evaluate the climate for change

In this step, Strategists and Implementers (*defined in Mento’s Step 2*) must unreservedly cognize how the organisation functions in its milieu, how it functions, and what its strengths and weaknesses are. This will enable constructing an effective implementation plan. To disseminate this step, we (course director of MBPG, the lead instructor of MBPG and the digital education advisor) performed a strengths (S) and weaknesses (W) opportunities (O) and threats (T) SWOT Analysis (Table 5). The SWOT analysis helped us to strategize the subsequent steps of the change plan.

**Table 5:**
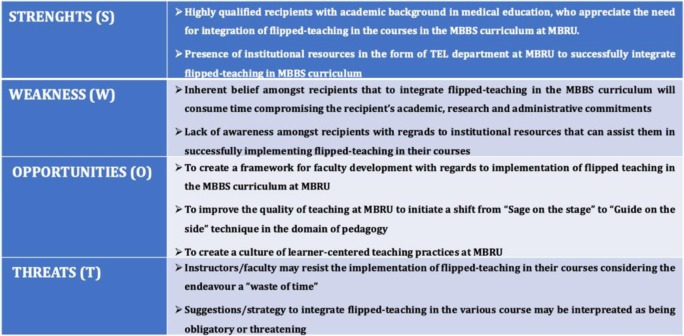
SWOT analysis

#### Mento’s step 4: develop a change plan

This step aims to craft an implementation plan to initiate the change. This plan should include specific goals, and provide detailed and clear responsibilities for strategists, implementers and stakeholders. For our project, the following implementation plan was strategized:

1. Strategists and implementers meet and discuss the suitability of the current institutional resources to implement flipped-teaching at MBRU. These include:
  - Suitability of teaching venues
  - Availability of the equipment to facilitate the design of teaching material associated with the delivery of course content through flipped-teaching.
  - Availability of web-resources where the developed teaching material can be hosted.
2. Delineate a faculty development plan such that concerned stake-holders can be informed with regards to various aspects of flipped-teaching.
3. Identify ways to assist stakeholders to implement flipped teaching in their courses.

#### Mento’s step 5: find and cultivate a sponsor

This step tracks to Kotter’s idea of developing a powerful guiding coalition and Jick’s view of lining political sponsorship (Fig. 1). In line, the overall idea of this step is to identify and cultivate individual(s) in one’s organization who will legitimize the change effort. In the present scenario we, identified the academic leadership at MBRU as our sponsor.

The strategists and the implementers of this project organized discussion sessions with the academic sponsors: A. Dean of the School of Medicine; B. Associate Dean of Education for the School of Medicine and C. Chairs of both Basic and Clinical Sciences; and presented to them the need for amending teaching strategies at MBRU.

The academic leadership was convinced that flipped-teaching was a learner-centred approach, which if implemented will enrich the academic experience of students at MBRU. In line with their assertion to support the initiative i.e. the idea of implementing flipped-teaching, it was proposed that a directive be disseminated and circulated among the faculty members at MBRU, directing the course-directors and instructors to actively support the initiative by integrating active-learning pedagogical techniques, specifically flipped-teaching in at least 40% of the teaching sessions. Additionally, it was also suggested that this implementation process be actively evaluated through peer-evaluation of teaching in specific courses in Phase-1 of the curriculum.

#### Mento’s step 6: prepare your target audience, the recipients of change

According to Mento any change isn’t possible unless people are willing to change themselves. However, whatever is the nature of change (positive or negative) resistance will always be encountered by implementers. This is because people are comfortable with knowns and the introduction of change is an unknown, which adds stress to recipients/stakeholders [37]. In line, a well-defined strategy should be in place to prepare the recipients of change. *In Mento’s Step 4*, a faculty development plan was delineated.

In this step we executed the faculty development plan (Table 6). In order for proper dissemination of the plan we designed short nano-lectures, catering to different AL teaching techniques. Although, in this study we have specifically focussed on the exemplar of flipped-teaching, we have also shared the nano-lectures for the other AL teaching techniques, which can be implemented in any CBME curriculum the employing the framework delineated in this study.

**Table 6:** Faculty development program

**Stage 1: A pre-recorded nano-lecture on flipped-method of teaching will be circulated among the stakeholders one-week prior to the faculty development program, using a WhatsApp group already in place. A nano-lecture is a 2 to 5 min lecture that is far shorter than and focuses on the key aspect of a specific topic. The video that we will use in this project (along with other videos) can be accessed in the Supplementary information.**
**Stage 2: Four days prior to the faculty development program a message will be sent to the stakeholders to record a short-video in style of a nano-lecture (based on what they learnt in Stage 1), which they may use for teaching. The stakeholders need to submit this video three hours prior to the commencement of the faculty development program.**
**Stage 3: All submitted videos will be posted on Padlet (****https://padlet.com****), which is a mobile application to create an online bulletin board, three hours prior to the faculty development program.**
**Stage 4: Faculty development program of 60-min duration. The following outline will be followed:** ➢ **Homework assessment – Viewing of the posted videos in Padlet.** ➢ **Review of submitted video – evaluation, feedback, and voting for “MBRU Oscar for best video”** ➢ **Discussion of implications of flipped-method of teaching on providing for in-class application of knowledge** ➢ **Discussion of flipped method of teaching approach focusing on advantages/disadvantages** ➢ **Case discussion: Critical appraisal of the study by Lichvar et al (Lichvar, Hedges, Benedict, & Donihi, 2016)** ➢ **Individual reflection regarding potential use of flipped-classroom pedagogy** ➢ **Small- and large-group discussion** ➢ **Evaluation of faculty development program**

*Following the execution of the faculty development plan, most of the stakeholders were supportive of integrating flipped-teaching in their courses. At the conclusion of the faculty development session, we had an informal feedback session where we deliberated on the PROS and CONS of implementing flipped-teaching (*Table 7*)*. We took note of the issues and decided to convey them to the sponsors.

**Table 7:**
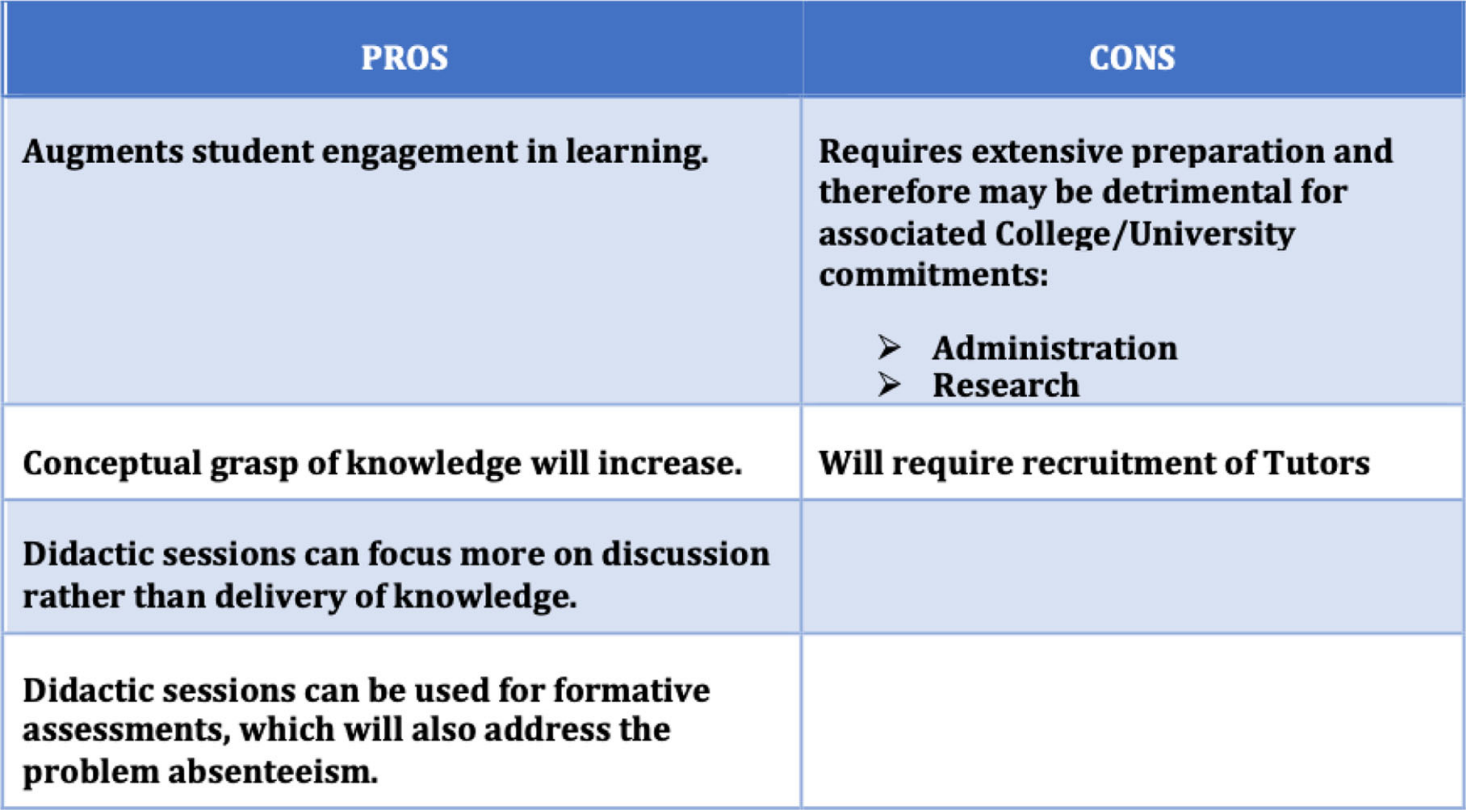
Stakeholder appraisal of flipped teaching

#### Mento’s step 7: create the cultural fit — making the change last

According to Mento, “*During the evolution of any change effort, the change must become rooted to the existing organizational culture*”, which will “make the change last” [37]. This project will avail a specific set of strategies to sustain the change, as shown in Table 8. Again, these strategies are generic and therefore can be adapted by any medical school offering a CBME curriculum.

**Table 8:** Strategies for making the change last

➢ Request the Sponsors for inclusion of a scoring scheme for implementing innovative pedagogical techniques in yearly faculty appraisal form.
➢ Appeal to the Department of Institutional Strategy at MBRU to allocate emoluments in form of funds to support faculty training in innovative teaching techniques including flipped model of teaching.
➢ Demonstrate to the faculty members through faculty development workshops, how implementation of flipped-model of teaching can lead to better student performance, as most of our current students are deep-learners and favour flipped-model of teaching. (*Akhras* et al.*, unpublished observations*)

#### Mento’s step 8: develop and choose a leader team

Unlike Kotter’s model of change [1, 32], Mento’s change model [37] proposes the formation of Leader Team. Such a team can be assembled to maximize the appropriate skill-sets. In the present project a Leader Team was formed, which consisted of *A. The course director of Biochemistry: myself; B. The lead instructor of Biochemistry; and C. The digital education advisor from TEL department.* The rationale for organizing such a team was, between us we had the required experience and the technical know-how to facilitate and guide the implementers to implement flipped-teaching in their courses. A leadership strategy was delineated by the Leader Team for functioning.

**Fig. 5:**
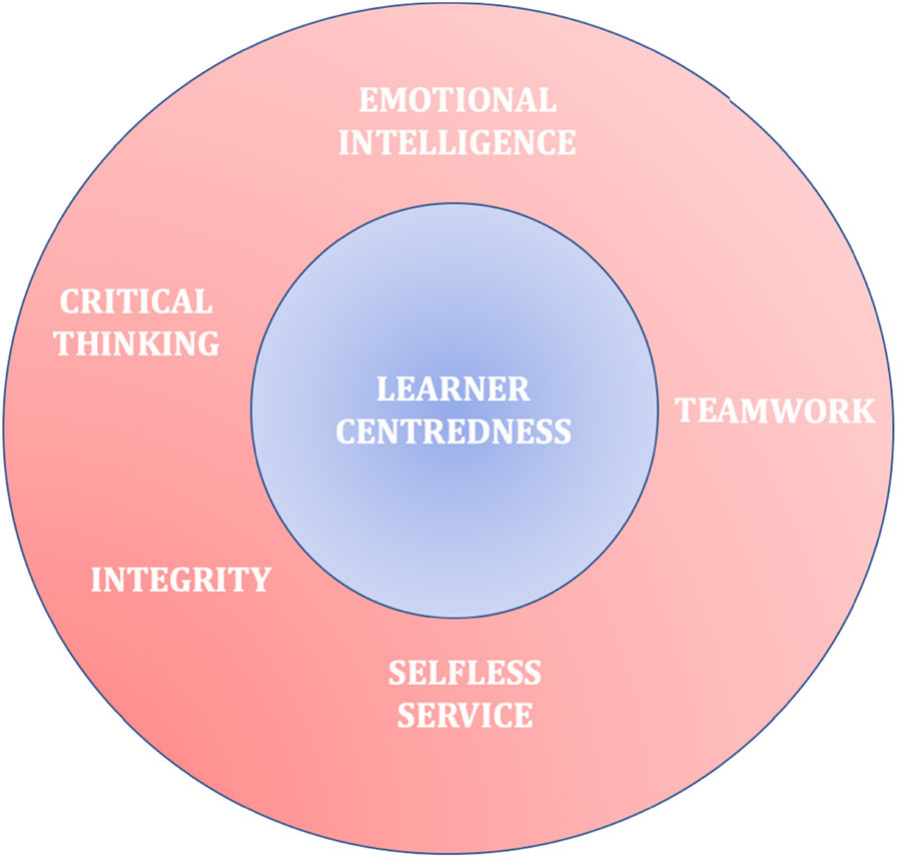
Leadership Model Adopted by The Leader Team. *(Note: We adapted The Duke Healthcare Leadership Model, substituting Patient Centredness with Learner Centredness)*

For the functioning of the Leader Team we developed a graphical representation of a shared leadership model in line with The Duke Healthcare Leadership Model (Fig. 5) [61]. The Duke Healthcare Leadership Model is developed using concept mapping and is based on the core principle of Patient Centeredness and core competencies of Emotional Intelligence, Integrity, Selfless Service, Critical Thinking, and Teamwork. In our, study we substituted patient centeredness with learner centeredness, but conserved the core competencies as these competencies have been identified by rank-sorting of 33 competency statements that represent important aspects of healthcare leadership by a diverse group of participants in the study which delineated The Duke Healthcare Leadership Model [61]. Adaptation of this strategy helped the Leader Team to identify the following challenges and adapt the guidance plan of change implementation accordingly.

First, being a proof-of-concept study, the time-frame of the project is relatively brief, which may pose a hurdle to the knowledge gain of the concerned stakeholders. Therefore, to address this impediment the successful implementation of the guidance plan involved the creation of a “Learning Community” (*Refer to Mento’s Step-10*), where stakeholders will learn from each other on the various aspects of flipped-teaching. Additionally, stakeholders who have successfully implemented the flipped model will discuss their experience in the Learning Community (*Refer to Mento’s Step-9*). Further, the Leader team will guide the stakeholders in their endeavour as well (*Refer to Mento’s Step-8*).

#### Mento’s step 9: create small wins for motivation

Stakeholder motivation is key during a change effort. Kotter has identified that one such way of generating and sustaining motivation during a change effort is “creating short-term wins” [32]. The idea of “creating short-term wins” is to recognize stakeholders who participate in/implement the change effort actively. One of the measurable goals of this project is to recognize faculty members who have implemented flipped-teaching in at least 40% of their teaching sessions. Faculty members who successfully achieve this goal will be:

A. encouraged to present their data/experience at faculty meetings at MBRU and at medical-education conferences
B. acknowledged of their success in the monthly university newsletter
C. offered tangible rewards in the form of best-teacher award
D. praised publicly for their contribution to the change effort e.g. in the faculty WhatsApp group, and will be requested to share their experience in the group

The above activities will also encourage the “so-called reticent” faculty members to also join the change effort. In line, the Leader Team have had discussions with the Sponsors with regards to supporting these initiatives, to which the Sponsors have responded positively. Additionally, the Sponsors are working towards integrating these aspects into faculty appraisal and faculty promotion guidelines, which will further cater to the success of the guidance plan.

#### Mento’s step 10: constantly and strategically communicate the change

This step tracks from Jick’s step 9 — ‘Communicate, involve people, and be honest’ [38] (Fig. 1). In this project the focus will be on communication with the sponsor, as well as to establish a bidirectional communication channel between the and the strategists and implementers who control the required resources.

The key focus in this project will be communication with the academic leadership (sponsors), who will be updated on a regular basis with regards to the progress of the change effort. Doing so will ensure the allocation of suitable resources/support to Stakeholders (faculty members) such that they can effectively implement flipped-teaching in their courses. Further, since MBRU is still in its formative years, if the sponsors deem that existing resources are underprovided to support stakeholders, they can collaboratively seek support from matured academic institutions. This endeavour will sustain the change effort unhindered. Case in point, initiation of Centre for Outcomes and Research in Education (CORE) at MBRU with guided support from McMasters University of Canada has expedited curricular innovation, shaping and reshaping competencies, testing new teaching/learning methods [62].

Additionally, through-out the change effort, the focus will be to:

A. create a “Learning community” such that stakeholders can learn from each other about strategies to successfully implement flipped-teaching in pedagogy.
B. try to address hurdles that are faced by stakeholders in their endeavour.

This will further ensure that change-effort is sustained and milieu for motivation is created.

#### Mento’s step 11: measure progress of the change effort

According to Mento “This step is in concurrence with step 6 of GE’s Change Acceleration Process, which is Monitoring Progress. This involves creating and installing metrics to assess programme success and to chart progress, using milestones and benchmarks” [39].

In this project the following will be availed to measure progress of the change effort:

A. Refer to the updated pedagogical techniques of concerned courses to appraise the number to teaching sessions where flipped-teaching was implemented.
B. Evaluate the attitude of stakeholders towards flipped-teaching following the transformation initiative using ADKAR framework [63], a questionnaire has been designed for this purpose.
C. Assess the performance of the students in these courses to identify if flipped-teaching was beneficial over traditional method.
D. Conduct student feedback to assess the perception of students towards flipped teaching, for which we have developed a 10 – item Likert scale questionnaire (*Appendix, Questionnaire I*).

#### Mento’s step 12: integrate lessons learned

Unlike other models of change, this step is unique to Mento’s change model. At the core of lessons learned is “***reflection***”. “Reflection is a personal cognitive activity that requires stepping back from an experience to think carefully and persistently about its meaning through the creation of inferences” [64].

### Reflecting on the guidance plan implemented in this project

#### Choosing the framework for reflection

Before penning our reflective piece, we had some difficulty in choosing a framework to abide by while drafting our reflection. Our first notion was to follow the widely accepted Kogan’s [65, 66] or Gibb’s [67] models of reflection, however, both these models looked overtly complex (when we wanted to apply them to our study). In line, we wanted something that felt simple, in a way such that we will have a rough-framework but will have the flexibility to direct our reflective-piece as we wanted the reader to capture the essence of the guidance plan.

Hence, we decided upon Gravin’s framework [68], which has also been adapted by the US Army in their After-Action Review process. The framework is centred on a set of FOUR questions:

1. **What did we set out to do?**
2. **What actually happened?**
3. **Why did it happen?**
4. **What are we going to do next time?**

#### What did we set out to do?

## Background

### Before we address the first question of Gravin’s framework let us first elaborate upon “*WHY WE SET OUT TO DO WHAT WE SET OUT TO DO?*”

At MBRU the curriculum is divided into three-phases, spanning over six-years [59]. Phase-1 of the curriculum aims to deliver the corpus of basic science courses, which should inform clinical reasoning and practice in the later Phases of the curriculum. This requires both horizontal, vertical and longitudinal integration in the formal curriculum [69–72]. However, a recent informal curriculum review, especially of the Phase – I of the curriculum, at MBRU showed that although there is contextualization (demonstrating the applicability of a basic science principle or concept in a clinical situation) [73], of basic science concepts, there is a dearth of integration. The key reason cited by the instructors for the dearth of integration was “paucity of active-learning practices to promote learner-centred education”. Learner-centred education “is part of a wider trajectory of curricular and pedagogical reform in higher education, “has its roots in constructivism and context-based theories,” and places emphasis on learning communities, integration, diverse pedagogies, and learning outcomes” [11]. One way to augment learner-centred education is to move away from the traditional didactic method of teaching and adapt pedagogical strategies which promote on-site knowledge assimilation.

#### The actual task

The lead author of this manuscript being the director of Phase-1 (Fig. 3) [59], was assigned the task of implementing learner-centred pedagogical strategies in the Phase-1 courses, for which the flipped-teaching pedagogical model was selected, as implementation of this teaching-model in MBPG showed positive outcomes [60].

### What actually happened?

#### Preparing for the task

First, the content of all the courses in Phase-1 were by a team of experts forming part of the curriculum committee. Based on this review appraisal it was decided that pedagogical model of flipped-teaching will be implemented in selective courses, specifically the structure-function courses (Table 3), as

A. they are content heavy.
B. the delivered concepts in these courses inform clinical reasoning to a greater extent in later Phases.

#### Transactional and transformational leadership, assembling of the leader team and assigning responsibilities

In order to decide on the members of the Leader Team (*Mento’s Step 8*), two benchmarks were considered:

A. Prior-experience with flipped-teaching
B. In-depth knowledge with regards to the technicalities required to implement flipped-teaching.

In line, the Leader Team consisted of the course director of MBPG and the lead-instructor of MBPG (the Strategists). The Leader Team also included the digital education advisor from TEL department of MBRU, who had in-depth technical know-how regarding the ‘nuts and bolts’ of the resources required to implement flipped-teaching in these courses (the Implementer). The Strategists were responsible for identifying the needs of the stakeholders with regards to them implementing flipped-teaching in their courses. The implementer on the other hand saw to the resources required to implement flipped-teaching. This shared leadership allowed multi-tasking as well as integrate constructive suggestions into the guidance plan.

#### How the leader Team’s leadership links theory to practice?

The Leader Team functioned on the Full Range Leadership Model (FRLM). In this model, a definite set of leadership apparatuses is essential for effective leadership: a sizeable measure of transformational leadership; advanced levels of transactional leadership and a minimum of Laissez Faire type leadership (Fig. 2). Transformational leadership refers “to leaders with an appealing vision for their team who intellectually stimulate others in a way that is demanding and appreciative of the individual needs of the team members” [36]. This is best demonstrated by the Leader Team’s ability garner support from both sponsors and stakeholders, with regards to implementation of flipped-teaching in the courses. Transactional leaders “exert influence on followers based on exchanging benefits for outstanding performance and response to their self-interests when they have achieved defined goals” [36]. This is best confirmed in this project where the Leader Team was able to convince the sponsors to “create small wins” for the stakeholders on successful implementation of flipped-teaching in their courses. Although, at all times the Leader Team has had a bird’s eye-view of the progress of the project and in the form of the faculty development supported the stakeholders to implement flipped-teaching, it never micromanaged or enforced any particular methodology of how to implement flipped-teaching or transgressed on stakeholder’s area of expertise, a classical exemplar of Laissez Faire type leadership [36].

#### Creating the Communication Channel

Communication is central to implementing change [74]. The Leader Team communicated the need for change to the stakeholders through the creation and dissemination of videos on flipped-learning, where the concluding section of the video presented the viewer with a concept map, such that the viewer can quickly grasp the importance and benefits of flipped-teaching. Additionally, our faculty development program was a big-success as majority of the stakeholders agree to implement flipped-teaching in their respective courses. Additionally, on request of the stakeholders a discussion group was created such that stakeholders and the implementers could effectively communicate. This allowed the stakeholders to learn from each other in line with principles of peer-assisted-learning [75], also helped the leader team to identify perceptions of stakeholders, as well as the opinion-leaders in the team, in guidance with the principles of network-theory [76].

#### Observed outcomes

Any change takes time. Currently this project is in progress, but what is important is that we have been successful in initiating a change, which is and will progress according to a defined guidance. Often masterminds implementing change get lost in the so-called imbroglio of multiple thoughts and theories, but in this project our plan to implement change is following a demarcated guidance plan strategized employing a validated change management model. This is the reason; we have achieved several milestones:

A. The Leader Team has been able to develop and disseminate a successful faculty-development program in collaboration with several stakeholders who now are “converted” and willing to implement flipped-teaching in their courses.
B. Further, as we want to implement change in an organized approach, we have experienced a collegiate environment as described by Hargreaves [77] as a setting of consensual, shared decision making; in which we found ourselves heard both by the Sponsors and Stakeholders. This allowed us to further seek both logistical and financial support for the project.
C. Our SWOT analysis (Table 5) showed that some of the Stakeholders may feel threatened with our change-effort. Indeed, we found resistance from specific stakeholders who believed the traditional method to be a superior compared to flipped-teaching. Indeed, this isn’t uncommon, as concerned stakeholders often resist organizational change [78]. However, implementation of our guidance plan is in several stages, therefore, we believe that through creation of small-wins and support from Sponsors such resistance can be effectively addressed in the long-run.

### Why did it happen?

#### The positives with a focus on emotional intelligence

Till now the project has yielded positive results. Our faculty development plan was critically appraised by our sponsors and found to be in line with our change efforts, so much so that the sponsors believe that is should be introduced as a faculty development program especially for faculty members who are participating in later phases of the curriculum. Also, because of shared leadership, we have been able to design not only an action plan for the project but prepare all the necessary materials and resources required to implement flipped-teaching in designated courses.

Further, we were able to identify a leadership strategy in lines with The Duke Healthcare Leadership Model [61] for our Leader Team to function. This model helped us to factor in the Emotional intelligence of both the Leader Team members and the stakeholders, assisting us to come up with effective guidance plan, and well as a strategy for creating small wins for motivation. In fact, works of Skinner and Spurgeon show that emotional intelligence plays a predominant role in healthcare leadership as it helps the leadership to understand and communicate effectively with diverse individuals in different situations, not just concentrating on outcomes and cogent processes [79].

#### The negatives

Although, most Stakeholders attest to our change-efforts, a few still aren’t convinced (based on initial informal discussion and WhatsApp threads). These so called “non-converts” are of the opinion that traditional didactic teaching has been in place for centuries, and a sudden transition from “traditional to flipped” isn’t going to make a significant different with regards to student-learning. Reflecting back this can be considered as a failure of the Leader Team. Although we expected resistance and took measures to tackle it, however, we fell short on specific aspects which need to be tackled (Refer below).

### What are we going to do next time?

#### Tackling the negatives

We believe that specific stakeholders aren’t convinced about our change-plan as they are unable to appreciate the bigger picture. Medical education in the twenty-first century requires to evolve according to the tenets of Flexnerism or in other words incorporate the teaching of basic sciences with clinical skills [59]. This aspect we believe was not adequately addressed in our faculty development plan, which we discounted as our SWOT analysis showed that concerned Stakeholders are highly qualified with background in medical education (Table 5).

Aarons et al. have shown that communication and collaboration are at the centre of change leadership practices [80]. Yes, till now we have adapted a shared leadership strategy and initiated a learning community, but on critically reflecting on the project so far our focus has been more towards addressing the needs of stakeholders who attested to the change-plan, but we may overlooked the “non-converts”. However, as the project progresses, by garnering support from the Sponsors and by communicating the need for integration of active learning strategies in the curriculum, we believe that the “non-converts” can be transformed to “converts”.

## Conclusions

ME is rapidly evolving, where “competency” is the buzzword, which has prompted amendments/reviews of the existing curricula disseminated in medical schools around the globe. Although most medical schools boast of administering competency based medical curricula, the ground reality is different. One of the key aspects of delivering CBME is to adapt AL pedagogical strategies. But few schools have been successful in this domain. When one tries to analyse the reason behind this “lack of success”, the foremost reason is intransigence of educators to “CHANGE the way they teach”. Coupled to this obstinacy is the dearth of information regarding the benefits of active pedagogical strategies.

*Take for example, one of the authors was conducting a one to one informal consultation with a senior faculty member in a medical school. This faculty member apart from being responsible for delivery of content in both basic and clinical phases of the medical curriculum, was also responsible for empowering junior faculty to improve and transform teaching techniques in the curriculum. When asked about how he provided feedback to the student, his reply was not only inaccurate but what was surprising was the fact that he didn’t want to mend his method to avail the correct one.* This shows that to initiate and implement change a well-designed guide plan is required, such that not only the initiation of change is successful, but patrons adapting to the change understand the need for change, and are concurrently provided with the knowledge required to implement the change and adapt to it.

In this manuscript, we present a framework for implementing change in medical education. In designing our framework, we have used Mento’s model of change, which was developed and successfully implemented in a fortune 500 defence industry firm, with positive outcomes. To elaborate on our framework, we have used a project (which is currently in progress) where the aim is to implement active learning strategy in the form of flipped-teaching in selected basic science courses in the medical curriculum. *The framework is not only supported by acknowledged leadership theories but can be translated to implement any curricular change in CBME.* In fact, our initial results are positive, which shows the versatility of Mento’s change model. We also present the readers, with a simple blueprint for reflection such that change process can be sustained with augmented benefits.

Lastly, the key aspect of initiating a change process is to communicate the urgency of change to the concerned.

Take for example, *when we tried to first implement the change process, we organized discussion-sessions with the concerned Stakeholders. Several of these sessions were organized, where we will talk in big-picture terms, key transformation issues and the vision of how to bring a change in teaching style* i.e. *to implement flipped-teaching. We indeed worked very hard at this. Soon these sessions, assumed a so-called monotone and Stakeholders lost interest. We’d give ourselves a 100 out of 100 for the effort, and a big ZERO for results. As the semester started to creep in, we realized we wouldn’t be able to implement flipped-teaching if we didn’t reorient our approach. Therefore, instead of saying “Let’s try to implement some change in teaching style in your courses”, we’d say “We are receiving extremely poor feedback on teaching from students in some of the courses in Phase-1, and if we do not address this, it may snowball into a disaster”. This tactic got everybody’s attention, both Sponsors and Stakeholders, following which implementation of our guide-plan became a cinch. What this taught us is that when alligators are nipping at your heels, one first needs to deal with the alligators, the big picture and the vision can wait! A tactic similar to that of Lou Gerstner when he took over IBM as its CEO, best surmised in his famous quote “The last thing we need now is a vision”*.

## Limitations and future directions

Although we have been successful in initiating change, employing Mento’s 12-step change management model, one of the key limitations of our study is that we are unable to provide concrete observations with regards to specifically the outcomes of the change process. This is because any change takes time to bear definite outcomes. Moreover, this is a proof of concept study where the foremost focus was to delineate a coherent framework to implement change, such that the defined framework can be adopted by any competency based medical curriculum in any medical school around the globe to initiate and sustain a specific and positive change.

Initiating change in medical education is not an easy feat. Medical schools are well known for their professional bureaucratic nature, in specific resistance to change. This can be designated as behavioural apathy, which is the propensity to preserve the prevailing organizational structure, even when it is evidently ineffective and unsuitable to legitimate goals. This is prevalent within medical schools globally, in the silhouette of an amalgamation of organizing practices, which are traditionally located and quixotically resistant and resilient. Behavioural apathy can affect the ability of a medical school to efficaciously and successfully implement a change in the medical curriculum, creating resistance to any constructive modification of existing practices. Even if a proposed change in the medical curriculum is supported by most stakeholders, there are a plethora of factors that play a role in how well the change is recognized, implemented and sustained. As identified in this study, those factors are resistance against change, internal communication on change, empowerment and involvement and organizational culture. In line bringing change to the niche of medical education specifically to the medical curriculum requires adequate preparation and ground settings in which change can be implemented and accepted. The primary focus of this study was to employ Mento’s change-management model to define and elaborate on a framework for change. The 12-steps inherent to this model tackle the key factors opposing change i.e. resistance against change, internal communication on change, empowerment and involvement and organizational culture.

Outcomes pertaining to individual steps must be suitably assessed using defined tools, which will form the basis of future studies. In fact, prior to this we designed the novel 6D-approach of Flipped Teaching [60], which when implemented in a specific course in the preclinical phase of medical curriculum and provided us with favourable outcomes. So, in the next phase of the study we wanted to translate flipped teaching in other courses of the pre-clinical phase of the medical curriculum. However, doing so required us to strategize a rational framework for which we employed a change-management model. Here we have elaborated on the theoretical background behind the approach, elaborating on the suitability of the model, the availed leadership approach and the way in which we were able to convince most of the concerned stakeholders with regards to the need for change in pedagogy. We firmly believe that the current framework will allow readers to blueprint strategies (with minor tweaking according to the need of the nature of change) to initiate and sustain any positive change in a competency based medical curriculum.
